# A Uniform Design Method Can Optimize the Combinatorial Parameters of Antimicrobial Photodynamic Therapy, Including the Concentrations of Methylene Blue and Potassium Iodide, Light Dose, and Methylene Blue’s Incubation Time, to Improve Fungicidal Effects on Candida Species

**DOI:** 10.3390/microorganisms11102557

**Published:** 2023-10-13

**Authors:** Meixia Du, Feng Li, Yanwei Hu

**Affiliations:** 1 Guangzhou Institute of Pediatrics, Guangzhou Women and Children’s Medical Center, Guangzhou Medical University, Guangzhou 510623, China; dmx286386993@163.com; 2 State Key Laboratory of Virology, Wuhan Institute of Virology, Center for Biosafety Mega-Science, Chinese Academy of Sciences, Wuhan 430071, China; fli@wh.iov.cn

**Keywords:** uniform design method, antimicrobial photodynamic therapy, Candida, methylene blue, potassium iodide

## Abstract

The optimal combinatorial parameters of antimicrobial photodynamic therapy (aPDT) mediated by methylene blue (MB) with the addition of potassium iodide (KI) against Candida species have never been defined. This study aimed to optimize the combinatorial parameters of aPDT, including the concentrations of MB (X_1_, 0.1–1.0 mM) and KI (X_2,_ 100–400 mM), light dose (X_3_, 10–70 J/cm^2^), and MB’s incubation time (X_4_, 5–35 min) for three Candida species. The best MB + KI-aPDT fungicidal effects (Y) against *Candida albicans* ATCC 90028 (Y_Ca_), *Candida parapsilosis* ATCC 22019 (Y_Cp_), and *Candida glabrata* ATCC 2950 (Y_Cg_) were investigated using a uniform design method. The regression models deduced using this method were Y_Ca_ = 7.126 + 1.199X_1_X_3_ − 1.742X_1_^2^ + 0.206X_2_^2^ − 0.361X_3_^2^; Y_Cp_ = 10.724 − 0.867X_1_ − 1.497X_2_ + 0.560X_3_ + 1.298X_2_^2^; and Y_Cg_ = 0.892 − 0.956X_1_ + 2.296X_3_ + 1.299X_4_^2^ − 3.316X_3_X_4_. The optimal combinatorial parameters inferred from the regression equations were MB 0.1 mM, KI 400 mM, a light dose of 20 J/cm^2^, and a 5-minute incubation time of MB for *Candida albicans*; MB 0.1 mM, KI 400 mM, a light dose of 70 J/cm^2^, and a 5-minute incubation time of MB for *Candida parapsilosis*; MB 0.1 mM, KI 100 mM, a light dose of 10 J/cm^2^, and a 35-minute incubation time of MB for *Candida glabrata*. The uniform design method can optimize the combinatorial parameters of aPDT mediated by MB plus KI to obtain the best aPDT fungicidal effects on Candida species, providing a new method to optimize the combinatorial parameters of aPDT for different pathogens in the future.

## 1. Introduction

Recently, the number of Candida infections has increased worldwide [1,2,3]. Candida infections can disseminate to nearly every organ of the body and cause severe medical problems [4]. *Candida albicans* (*C. albicans*) continues to be the most prevalent [5,6], followed by *Candida parapsilosis* (*C. parapsilosis*) [7]. Meanwhile, *Candida glabrata* (*C. glabrata*) is the most common non-albicans Candida spp. involved in recurrent vulvovaginal candidiasis in China [8]. Given the limited classes of drugs available to treat Candida infections [9], as well as the increasing drug resistance of Candida species, the efficacy of these traditional agents is compromised [1,9].

Antimicrobial photodynamic therapy (aPDT) has been an alternative therapeutic approach to fight the growing problem of antimicrobial resistance that threatens health care [10,11,12]. aPDT involves the use of a non-toxic dye (also called a photosensitizer) excited with visible light to produce reactive oxygen species (ROS) that can destroy all classes of microorganisms, including bacteria, fungi, parasites, and viruses [10], even for multidrug-resistant pathogens [13]. Recently, some inorganic salts, with potassium iodide (KI) being the most commonly used, have been proven to be an effective adjuvant to enhance aPDT killing efficiency [14,15,16]. Many parameters can influence the effectiveness of aPDT, including the concentration of the photosensitizer, the light dose, the concentration of potassium iodide (KI), and the incubation time of the photosensitizer. Using these parameters appropriately is essential to produce the best aPDT fungicidal effects.

Methylene blue (MB) is a phenothiazinium photosensitizer commonly used in aPDT research [17,18], since it was one of the first clinically approved photosensitizers [19]. Ample studies have found that MB-aPDT is effective in killing Candida species [20,21]. KI has been proven to enhance the bactericidal effects of aPDT in previous studies [22,23], and it has been approved by the FDA for clinical use [24]. MB-mediated aPDT with the addition of KI (MB + KI-aPDT) was efficient in killing *C. albicans* in an immunosuppressed mouse model of oral candidiasis infection [25]. Our earlier clinical trial also found that MB + KI-aPDT is useful in treating oral candidiasis in AIDS patients [26].

Although MB has been used for aPDT research on *C. albicans* in plenty of studies, the survival rates of *C. albicans* cells vary from 0 to 100% when using the same photosensitizer (MB) and similar irradiation wavelength [27]. The distinctions found were in the concentrations of MB used, the incubation time, the illumination time, and the light dose, which were varied. However, the best combination of these aPDT elements has been ill-defined. Some scientists have found that the significant reduction in *C. albicans* cell growth is MB-concentration- and fluence-dependent [28], while others have found that increasing the MB concentration [26] or prolonging the duration of irradiation [29] does not improve the efficiency of aPDT on *C. albicans*. 

The concentration of KI is also vital. After adding KI, the photobleaching effect of MB could increase, thereby lightening the blue color of MB [22]. This alteration could also affect the quantities and species of reactive radicals. It has been proven that adding KI to MB-aPDT can increase the production of free iodine and hydrogen peroxide [22]. Previous research on synergistic mechanisms has shown that if the KI concentration used is relatively low (up to 10 mM), the iodine radicals are mainly responsible for killing the pathogens, while with an increased KI concentration up to 100 mM (or up to 400 mM), free iodine is mainly responsible [30]. Therefore, after adding KI as an enhancer of aPDT, the other aPDT parameters also need to be correspondingly improved. 

Hence, the best combinatorial parameters of aPDT are demanded. Whenever applying aPDT, it is crucial to carefully select appropriate concentrations of the photosensitizer and the enhancer, along with determining the photosensitizer’s incubation time and the light dose (which includes the energy and time of irradiation). Such combinatorial selection is essential for successful aPDT application. Nevertheless, research designed for optimizing the concentrations of MB and KI, incubation time, and light dose simultaneously to obtain the best aPDT killing efficiency has not been conducted.

Because there were at least four aPDT parameters, and each parameter had a lot of test levels, traditional design methods cannot find the best combination except by using the uniform design method. The uniform design method is an approach used to explore the optimal combination for multifactorial and multilevel experiments. This method allows for the automatic classification of test aPDT parameters as either important or minor, and the factors are ranked in order of importance based on the coefficient of correlation. It can also explore whether there are any interactions between these aPDT parameters that can affect the final aPDT fungicidal effects, which has been ignored in previous studies.

Furthermore, studies using MB + KI-aPDT against non-albicans Candida species are sparse. Hence, the objective of the present study was to optimize the combinatorial parameters of MB + KI-aPDT using the uniform design method on three Candida species (*C. albicans* ATCC 90028, *C. glabrata* ATCC 2950, and *C. parapsilosis* ATCC 22019). This research can help improve the efficacy of aPDT in prospective clinical applications. 

## 2. Materials and Methods

### 2.1. Chemicals and Reagents

Methylene blue (MB) was purchased from Sigma-Aldrich (St. Louis, MO, USA). Potassium iodide (KI) was purchased from Shanghai Yuanye Biotechnology Co., Ltd. (Shanghai, China). Phosphate-buffered saline (PBS, PH 7.4) was purchased from Shenzhen Biocomma Biotechnology Co., Ltd. (Shenzhen, China). MB stock solution and KI solution were prepared in laboratory water (distilled, deionized, and Milli-Q). MB stock solution was stored at 4 °C in the dark for no more than 2 weeks prior to use. KI solution was prepared as required immediately before experimentation.

### 2.2. Cells and Culture Conditions

The Candida strains used in the study were *C. albicans* ATCC 90028, *C. parapsilosis* ATCC 22019, and *C. glabrata* ATCC 2950 (Microbiologics. Inc., St. Cloud, MN, USA). Potato dextrose agar (PDA) and Sabouraud glucose agar with chloramphenicol (SDA) were purchased from Qingdao Hope Bio-Technology (Qingdao, Shandong, China). The three Candida strains were all first recovered on PDA three times according to the instructions, then preserved by suspension in 20% glycerol in small vials and stored at −70 °C until the day of the test. Before testing, the three Candida isolates were subcultured for 24 h on SDA at 35 °C, repeating the process three times. Stock inoculum suspensions of the yeast cells were prepared in sterile PBS. The turbidity of each yeast suspension was adjusted by spectrophotometry to a concentration of ~10^7^ cells per mL. The adjusted yeast suspensions before and after aPDT were inoculated on CHROMagar (CHROMagar^TM^ Candida, Paris, France) and SDA for 36 to 48 h at 35 °C. 

### 2.3. Light Source

**Figure 1:**
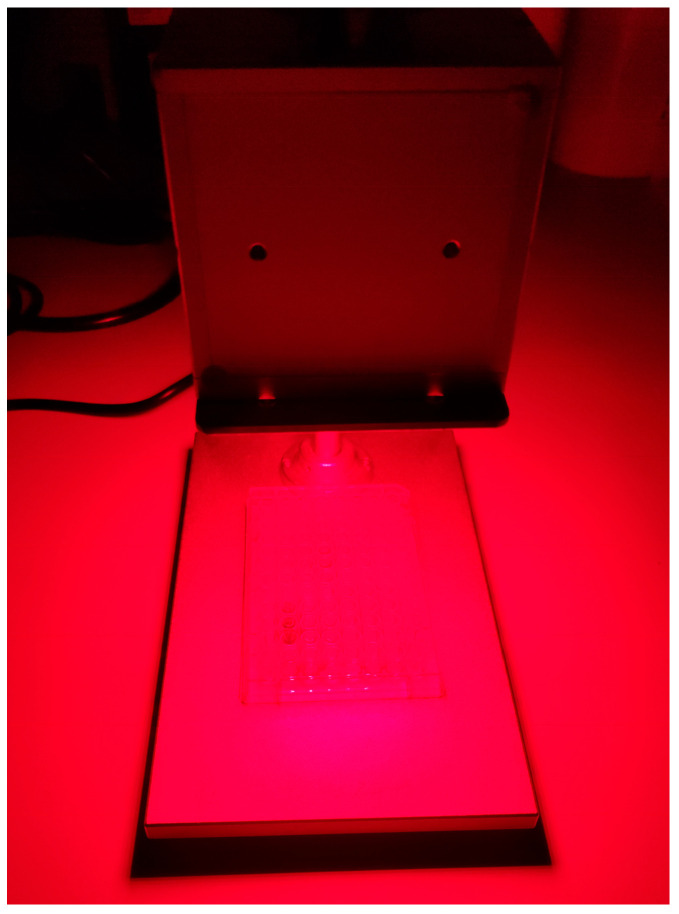
Photograph of aPDT during illumination.

The light source was a plane light-emitting diode (LED) device (Shenzhen Height-LED Opto-electronics Technology Co., Ltd, Shenzhen, Guangdong, China). The efficient light-emitting area was 100 × 50 mm, with a wavelength of 660 nm and a power of 50 mW/cm^2^ (Figure 1).

### 2.4. Study Design

The ranges of the four parameters were determined based on previous literature. As for the MB concentrations, they ranged from 0.3 to 60 mM in clinical MB-aPDT treatments [17], from 0.3 to 1 mM in MB-aPDT experiments on *C. albicans* [31,32,33], and from 0.1 to 0.6 mM in MB + KI-aPDT studies on *C. albicans* [25,26]. Therefore, a range of 0.1 to 1.0 mM was established for MB. KI concentrations of 200–400 mM strongly potentiated aPDT killing on Gram-positive (MRSA) and Gram-negative (*Escherichia coli*) bacteria [16]; concentrations of 100 to 300 mM were effective in enhancing the MB-aPDT killing effects on *C. albicans* [25,26], thus KI concentrations of 100–400 mM were selected. Light doses used in MB-aPDT ranged from 6 to 180 J/cm^2^, with the majority falling within the 10–70 J/cm^2^ range [25,26,27], which aligns with the range we selected. MB was incubated for various durations ranging from 1 min to 24 h in the MB-aPDT investigations. Among these durations, 5 min, 10 min, and 30 min were observed to occur frequently [27]. To comply with the U_7_(7^4^) uniform design method, we selected an incubation time for the MB of 5 to 35 min. The concentration of MB ranged from 0.1 to 1.0 mM; the concentration of KI ranged from 100 to 400 mM; the light dose ranged from 10 to 70 J/cm^2^ (with an irradiation time ranging from 200 s to 1400 s); and the incubation time for MB ranged from 5 to 35 min. These variables were divided into seven levels and distributed uniformly according to the U7(7^4^) uniform design method (Table 1). The U_7_(7^4^) uniform design method had a corresponding parameters assignment table (Table 2). There were seven schemes named A to G, which distributed the seven levels of the four parameters evenly. Schemes A to G employed MBs, KIs, light doses, and incubation times from the same rows of Table 2 during the experiments. For example, in scheme A, MB 0.4 mM was pre-incubated in the dark for 30 min, then KI 150 mM was added, and the solution was illuminated with a light dose of 30 J/cm^2^. Principles for the U_6_(6^4^) uniform design method were similar to for U_7_(7^4^), except that the ranges of the four parameters were different. 

### 2.5. aPDT Studies

#### 2.5.1. U_7_(7^4^) Uniform Design Method

Suspensions of each Candida inoculum (~10^7^ cells per mL) were incubated in the dark at room temperature for 5 to 35 min with 0.1 to 1.0 mM MB. A range of KI concentrations between 100 and 400 mM in pH 7.4 PBS was added. Then, they were illuminated with an energy density ranging from 10–70 J/cm^2^, depending on the irradiation time, which varied from 200 to 1400 s. The specific parameters in each experiment were represented by schemes A to G, as shown in Table 1 and Table 2.

#### 2.5.2. U_6_(6^4^) Uniform Design Method

Suspensions of each Candida inoculum (~10^7^ cells per mL) were incubated in the dark at room temperature for 50 to 100 s with 0.05 to 0.1 mM MB. A range of KI concentrations between 50 and 100 mM in pH 7.4 PBS was added. Then, they were illuminated with an energy density ranging from 5 to 10 J/cm^2^, depending on the irradiation time, which varied from 100 to 200 s. The specific parameters in each experiment are presented as schemes H to M, as shown in Table 3 and Table 4.

#### 2.5.3. aPDT Applications

Suspensions of each Candida inoculum (~10^7^ cells per mL) were divided into eight groups, four in the dark and four in the light. An aliquot of 100 μL was used for the dark control (DC), dark with MB (DM), dark with KI (DK), and dark with MB plus KI (DMK) from each sample. Another aliquot of 100 μL was used for the light control (LC), light with MB (LM), light with KI (LK), and light with MB plus KI (LMK) from each sample. These aliquots were transferred to a 96-well flat-bottomed plate (Corning Incorporated, Kennebunk, ME, USA) and illuminated from the top of the plates at room temperature using the aforementioned light device (Figure 1). At the completion of illumination (or dark incubation), aliquots (10 μL) were taken from each well to determine colony forming units (CFU). The contents of the wells were mixed thoroughly before sampling, as Candida inoculum can settle at the bottom. The aliquots were serially diluted 10-fold in PBS to give dilutions of 10^−1^ to 10^−4^ times in addition to the original concentration. Then, 10 μL aliquots of each dilution were streaked horizontally on square CHROMagar and SDA agar plates [34]. Plates were streaked in triplicate and incubated for 36–48 h at 35 °C in the dark to allow colony formation. Each experiment was performed at least three times.

Survival fractions were routinely expressed as the ratios of the CFUs of Candida cells treated with light, MB and/or KI (or MB and/or KI in the absence of light) to the CFUs of Candida cells in the DC groups. The regression models of MB + KI-aPDT and MB-aPDT efficiency were obtained with the U_7_(7^4^) and U_6_(6^4^) uniform design methods on three Candida species using log_10_(CFU_DC_/mL) minus log_10_(CFU_LM_/mL) or log_10_(CFU_LMK_/mL).

### 2.6. Statistical Analysis

Analyses, statistical tests, and figures were managed using SPSS software (version 22.0) and GraphPad Prism software (version 9.5). The aPDT parameters’ coefficients of correlation and regression 3D scatter plots were visualized using the function Pheatmap and scatterplot3d packages in R. The data were presented as either mean ± standard deviation or median with interquartile range, depending on the distribution of the data. The optimal combination of aPDT parameters, including concentrations of MB and KI, light dose, and the incubation time of MB, was investigated using the uniform design method. The killing efficiency of aPDT on Candida species and the parameters of aPDT were modeled using second-order polynomial equations. Relationships between the aPDT killing efficiencies on Candida strains and the aPDT parameters were examined by correlation coefficient. Significance was defined as a two-tailed *p*-value < 0.05 for all analyses.

## 3. Results

### 3.1. Survival Fractions of aPDT Schemes A to G on Three Candida Strains

**Figure 2:**
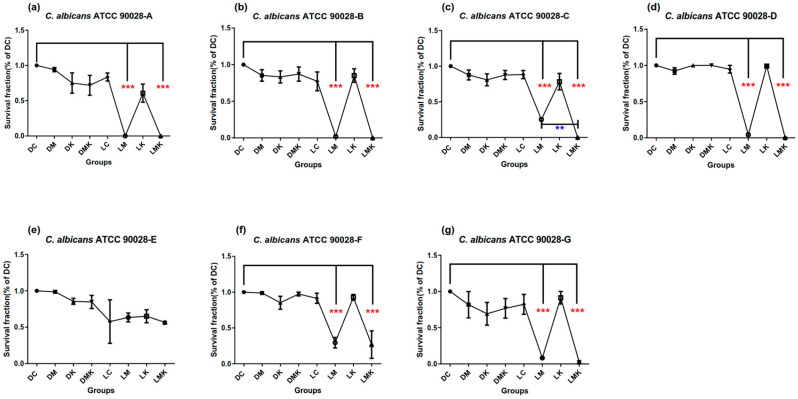
Survival fractions of aPDT schemes A–G (**a**–**g**) on *C. albicans* ATCC 90028. Notes: DC, dark control; DM, dark with MB; DK, dark with KI; DMK, dark with MB plus KI; LC, light control; LM, light with MB; LK, light with KI; LMK, light with MB plus KI. All schemes had DM, DK, DMK, LC, LM, LK, and LMK groups compared with their corresponding DC groups; LM and LMK groups showed significant differences except in scheme E (**e**); Comparing LM and LMK groups with each other in each scheme, only scheme C (**c**) showed significance; the red color of asterisks (*) in (**a**–**g**) represents significance when compared with the DC groups; the blue color of asterisks (*) in (**c**) represents significant groups compared with each other; *** represents *p* < 0.001; ** represents *p* < 0.01.

**Figure 3:**
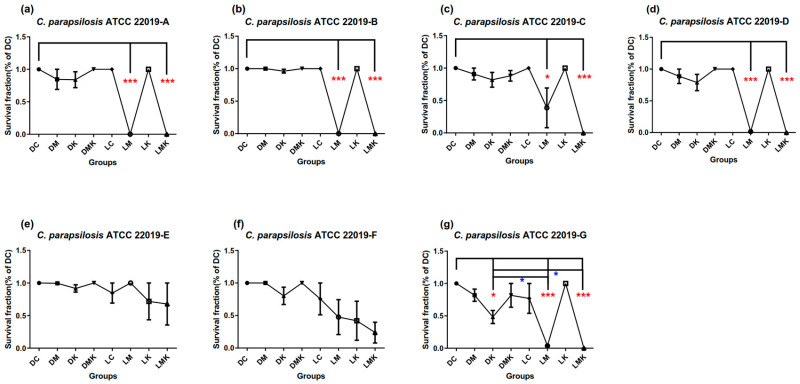
Survival fractions of aPDT schemes A–G (**a**–**g**) on *C. parapsilosis* ATCC 22019. Notes: DC, dark control; DM, dark with MB; DK, dark with KI; DMK, dark with MB plus KI; LC, light control; LM, light with MB; LK, light with KI; LMK, light with MB plus KI. All schemes had DM, DK, DMK, LC, LM, LK and LMK groups compared with their corresponding DC groups; LM and LMK groups showed significant differences except in schemes E and F (**e**,**f**); all DK groups showed no significant differences except in scheme G (**g**); in no scheme did the comparisons between LM and LMK groups show significance; in scheme G (**g**), the LM and LMK groups had lower survival fractions than the DK group; the red color of asterisks (*) in (**a**–**g**) represents significance when compared with the DC groups; the blue color of asterisks (*) in (**g**) represents significant groups compared with each other; *** represents *p* < 0.001; * represents *p* < 0.05.

**Figure 4:**
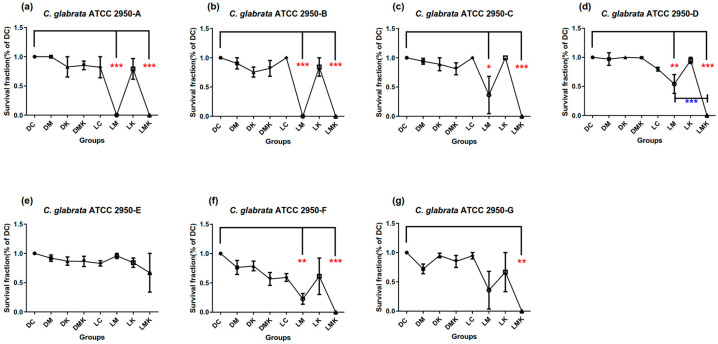
Survival fractions of aPDT schemes A–G (**a**–**g**) on *C. glabrata* ATCC 2950. Notes: DC, dark control; DM, dark with MB; DK, dark with KI; DMK, dark with MB plus KI; LC, light control; LM, light with MB; LK, light with KI; LMK, light with MB plus KI. All schemes had DM, DK, DMK, LC, LM, LK and LMK groups compared with their corresponding DC groups; LM and LMK groups showed significant differences except in scheme E (**e**); Comparing LM and LMK groups with each other in each scheme, only scheme D (**d**) showed significance; the red color of asterisks (*) in (**a**–**g**) represents significance when compared with the DC groups; the blue color of asterisks (*) in (**d**) represents significant groups compared with each other; *** represents *p* < 0.001; ** represents *p* < 0.01; * represents *p* < 0.05.

Survival fractions of aPDT schemes A–G on three Candida species are presented in Figure 2, Figure 3 and Figure 4. Among all Candida strains, the MB + KI-aPDT and MB-aPDT schemes from U_7_(7^4^) were all effective, except for scheme E (Figure 2e, Figure 3e and Figure 4e); Scheme F was effective for all strains, except for *C. parapsilosis* ATCC 22019 (Figure 3f). Other control groups (DM, DK, DMK, LC, and LK) in each scheme showed no significant difference compared with their DC groups, except in scheme G for *C. parapsilosis* ATCC 22019 (Figure 3g), which used a KI concentration of 400 mM. The effective MB + KI-aPDT schemes were superior to or equal to their corresponding MB-aPDT schemes in all three Candida strains. 

The regression models for MB + KI-aPDT and MB-aPDT efficiency, obtained using the U_7_(7^4^) uniform design method on three Candida species, are displayed in Table 5. The fungicidal efficiency of aPDT was defined as Y, and X_1_, X_2_, X_3,_ and X_4_ in the regression model obtained by the uniform design method represent MB concentration, KI concentration, light dose, and incubation time for MB, respectively.

**Figure 5:**
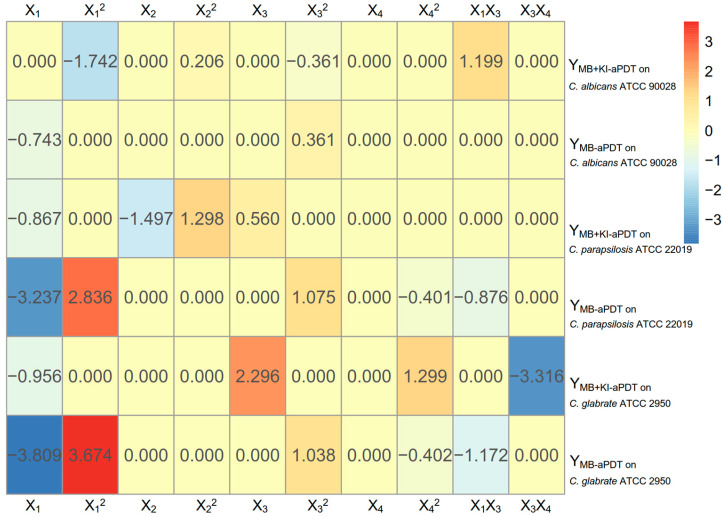
The regression coefficients of MB + KI-aPDT and MB-aPDT obtained by the U_7_(7^4^) uniform design method. Notes: aPDT fungicidal efficiency is defined as Y; X_1_, X_2_, X_3_, and X_4_ represent MB concentration, KI concentration, light dose, and MB’s incubation time, respectively.

The regression coefficients of MB + KI-aPDT and MB-aPDT obtained by the U_7_(7^4^) uniform design method are displayed in Figure 5.

**Figure 6:**
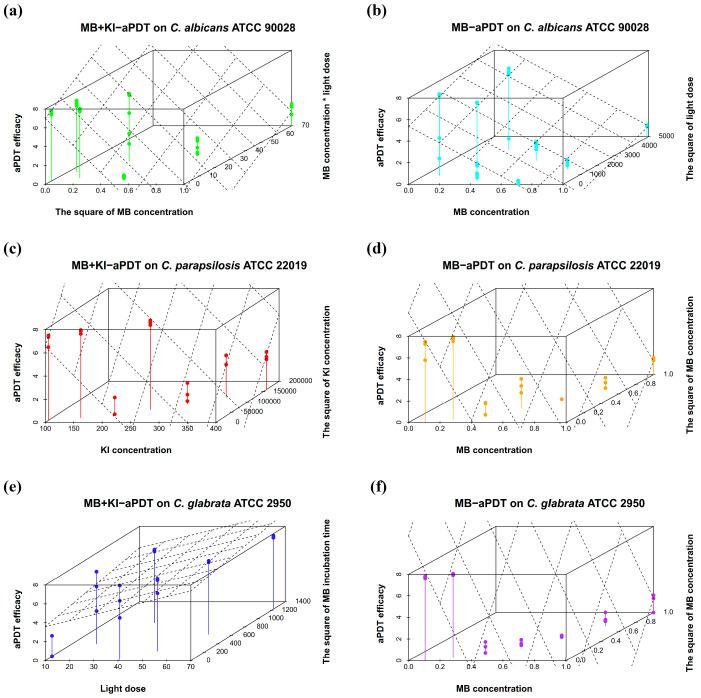
Regression 3D scatter plots of MB + KI-aPDT and MB-aPDT efficacy with associated parameters. Notes: Length, width, and height in each 3D scatter plot represent the x, y, and z-axes, respectively, with each z-axis representing aPDT efficacy. Planes represent the predicted values of aPDT efficacy, while dots represent the actual values. MB concentration * light dose on the y-axis in plot (**a**) represents interactions between MB concentration and light dose. (**a**,**c**,**e**) are regression 3D scatter plots of MB + KI-aPDT on *C. albicans* ATCC 90028 (**a**), *C. parapsilosis* ATCC 22019 (**c**), and *C. glabrata* ATCC 2950 (**e**), while (**b**,**d**,**f**) are regression 3D scatter plots of MB-aPDT on *C. albicans* ATCC 90028 (**b**), *C. parapsilosis* ATCC 22019 (**d**), and *C. glabrata* ATCC 2950 (**f**).

Figure 6 shows the visualization of different aPDT parameters to predict the efficacy of MB + KI-aPDT and MB-aPDT using multiple regression equations. These aPDT parameters include individual parameters, interactions between parameters, and squares of parameters, which are factors from the regression models presented in Table 5. The predicted values of aPDT efficacy are represented by planes, while the actual values are represented by dots. According to Table 5, some regression models have included more than two factors. However, 3D scatter plots cannot display all factors simultaneously; thus, each 3D scatter plot only exhibits two important factors that influence the corresponding aPDT efficacy. 

#### 3.1.1. Survival Fractions of aPDT Schemes A–G on *C. albicans* ATCC 90028

It was shown in Figure 2c that scheme C had a higher MB + KI-aPDT efficiency than MB-aPDT in killing *C. albicans* cells. Other effective MB + KI-aPDT schemes were equivalent to their corresponding MB-aPDT schemes.

According to the heat map of regression coefficients (Figure 5), within the U_7_(7^4^) uniform design range, the killing efficiency of MB + KI-aPDT on *C. albicans* ATCC 90028 was negatively associated with MB concentration (primarily) and light dose, while weakly positively associated with KI concentration. There were interactions between MB concentration and light dose, which were positively associated with aPDT killing efficiency on *C. albicans*. As for the killing efficiency of MB-aPDT, it was positively correlated with light dose but negatively correlated with MB concentration, with no interactions between the parameters. It is worth noting that neither MB + KI-aPDT nor MB-aPDT showed any relationship between their killing efficiency on *C. albicans* and the incubation time of MB.

#### 3.1.2. Survival Fractions of aPDT Schemes A–G on *C. parapsilosis* ATCC 22019

Figure 3g shows that 400 mM KI has a slight killing effect on *C. parapsilosis* cells, but it is less effective than MB-aPDT or MB + KI-aPDT application. The survival fractions in schemes E and F did not have significant differences compared with their respective DC groups or other control groups (Figure 3e,f).

According to Figure 5, within the range of the U_7_(7^4^) uniform design, the killing efficiency of MB + KI-aPDT on *C. parapsilosis* ATCC 22019 was negatively associated with MB concentration while positively associated with light dose. Notably, the KI concentration was nonlinearly associated with the killing efficiency of MB + KI-aPDT on *C. parapsilosis* ATCC 22019. In line with *C. albicans*, there was no relationship between aPDT killing efficiency on *C. parapsilosis* ATCC 22019 and MB’s incubation time.

With regard to MB-aPDT killing efficiency, it was positively associated with light dose but negatively associated with MB’s incubation time. There were interactions between MB concentration and light dose, and the coefficient of correlation was negative. The MB concentration was nonlinearly associated with aPDT killing efficiency.

#### 3.1.3. Survival Fractions of aPDT Schemes A–G on *C. glabrata* ATCC 2950

It can be observed that scheme D had a better MB + KI-aPDT efficiency than MB-aPDT in killing *C. glabrata* ATCC 2950 (Figure 4d). 

As shown in Figure 5, within the range of the U_7_(7^4^) uniform design, the aPDT killing efficiency on *C. glabrata* ATCC 2950 was positively associated with light dose and MB’s incubation time, while negatively associated with MB concentration and the interactions between light dose and MB’s incubation time. KI concentration seemed to have no relationship with the MB + KI-aPDT killing efficacy on *C. glabrata* ATCC 2950. 

However, the MB-aPDT killing efficiency was nonlinearly associated with MB concentration, positively associated with light dose, while negatively associated with the MB’s incubation time and the interactions between the MB concentration and light dose. 

### 3.2. Survival Fractions of aPDT Schemes H to M on Three Candida Strains

**Figure 7:**
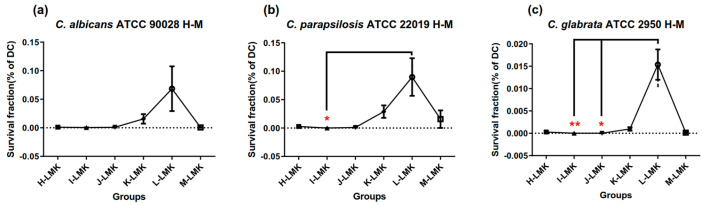
Survival fractions of aPDT schemes H–M on three Candida strains. Notes: LMK, light with MB plus KI; survival fractions of H to M schemes’ LMK groups were compared with each other in each Candida strain (**a**–**c**). (**a**) Survival fractions of H to M schemes on *C. albicans* ATCC 90028; LMK groups did not exhibit a significant difference in *C. albicans* ATCC 90028; (**b**) Survival fractions of H to M schemes on *C. parapsilosis* ATCC 22019; the LMK group in scheme L showed a significant difference from that in scheme I in *C. parapsilosis* ATCC 22019; (**c**) Survival fractions of H to M schemes on *C. glabrata* ATCC 2950; the LMK groups of schemes I and J exhibited significant differences from that of scheme L in *C. glabrata* ATCC 2950; ** represents *p* < 0.01; * represents *p* < 0.05.

According to the regression models mentioned above, within the range of the U_7_(7^4^) uniform design, the MB + KI-aPDT killing efficiency on all the three Candida strains was negatively associated with MB concentration. As is well known, the concentration of a photosensitizer is the most vital element in aPDT applications. Thus, a U_6_(6^4^) uniform design method was employed, assigned as H to M, to obtain lower ranges for MB concentration levels and the other three parameters. Since MB + KI-aPDT in the U_7_(7^4^) uniform design exhibited that control groups (DM, DK, DMK, LC, LK) with bottom levels did not show significant fungicidal effects on three Candida strains, and MB + KI-aPDT showed the best fungicidal effects, H-M schemes only focused on MB + KI-aPDT’s fungicidal effects (Figure 7).

**Figure 8:**
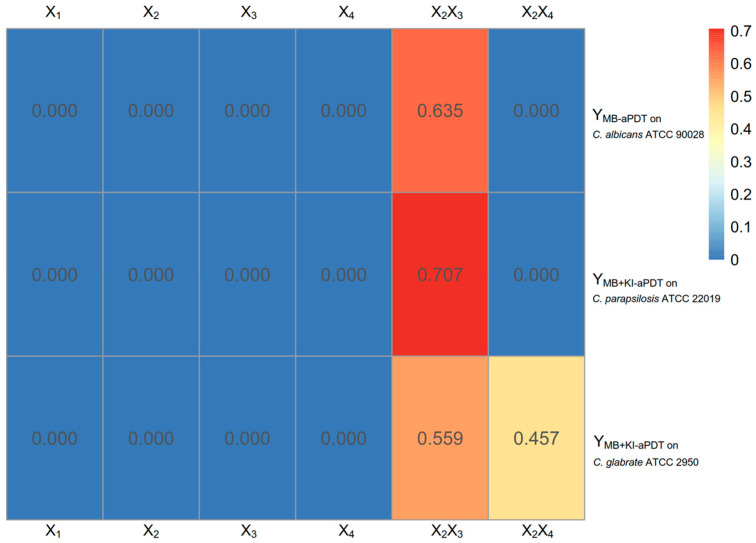
The regression coefficients of MB + KI-aPDT and MB-aPDT obtained by the U_6_(6^4^) uniform design method. Notes: aPDT fungicidal efficiency was defined as Y; X_1_, X_2_, X_3_, and X_4_ represent MB concentration, KI concentration, light dose, and MB’s incubation time, respectively.

Figure 7 demonstrates that scheme L had a significantly different aPDT efficacy in killing *C. parapsilosis* ATCC 22019 compared with scheme I (Figure 7b), as well as in killing *C. glabrata* ATCC 2950 compared with schemes I and J (Figure 7c). The regression models and coefficients are demonstrated in Table 6 and Figure 8.

According to Table 6 and Figure 8, within the range of the U_6_(6^4^) uniform design, the aPDT killing efficiency on three Candida strains had no relationship with MB concentration, but there were interactions between KI concentration and light dose, with or without interactions between KI concentration and MB’s incubation time. 

### 3.3. Results for Optimized MB + KI-aPDT Parameters

From a comprehensive perspective, the U_7_(7^4^) uniform design method exhibited a better model fit R than the U_6_(6^4^) method (Table 5 and Table 6). Additionally, within the range of the U_6_(6^4^) uniform design, the aPDT killing efficiency on three Candida strains was no longer related to MB concentration. As a consequence, the optimization of aPDT parameters on three Candida strains was established using the U_7_(7^4^) uniform design method. The optimized parameters for MB + KI-aPDT can be estimated based on the regression model equations provided in Table 5. Table 7 lists the combinations of optimized MB + KI-aPDT parameters. These parameters were selected by taking aPDT killing efficiency (Y), its economic effect, and clinical patient compliance into consideration together (Table 7). The optimized MB + KI-aPDT parameters show that three Candida strains had to employ the same bottom MB concentration (0.1 mM), but varied KI concentration, light dose, and MB’s incubation time to obtain best aPDT killing effects within the range of the U_7_(7^4^) uniform design. 

**Figure 9:**
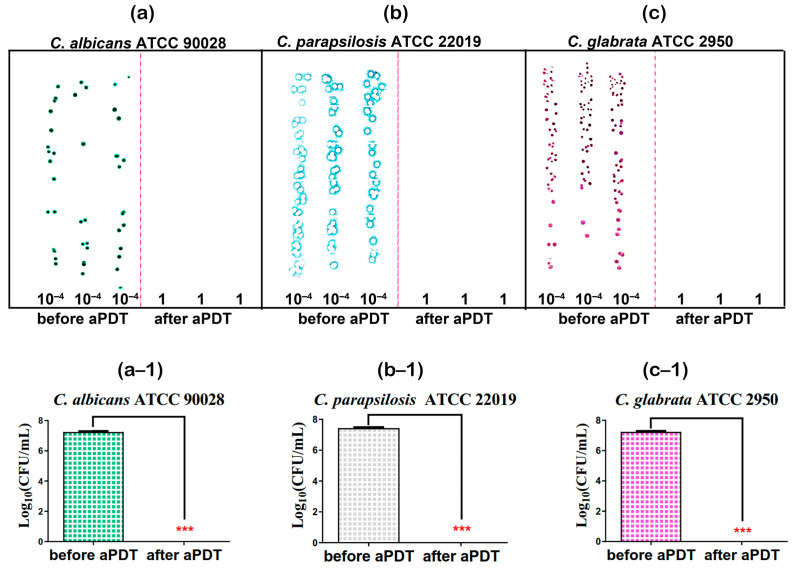
MB + KI-aPDT killing effectiveness of optimal aPDT parameters on three Candida strains. Notes: (**a**–**c**) are scanned images of viable Candida culture colonies on square CHROMagar before and after one session of MB + KI-aPDT killing; Candida cells were all eradicated after one session of MB + KI-aPDT. *C. albicans* ATCC 90028 (**a**) and *C. glabtata* ATCC 2950 (**c**), respectively, exhibited dark green and purple culture colonies on CHROMagar, whereas *C. parapsilosis* ATCC 22019 (**b**) showed white culture colonies which were not clearly visible in the scanned image; thus, a blue background color was added to highlight the culture colonies. A value of 10^−4^ means the aliquots were serially diluted to 10^−4^ times the original concentration, while 1 means no dilution from the original concentration. (**a−1**)–(**c−1**) are the statistical results of Log_10_ (CFU/mL) before and after one session of MB + KI-aPDT killing on three Candida strains, which was repeated at least three times; *** represents comparisons of Log_10_(CFU/mL) before and after aPDT, all *p* < 0.001.

We employed the optimized MB + KI-aPDT parameters listed in Table 7 on three Candida strains. The results verified that these optimized MB + KI-aPDT parameters could eradicate the three Candida cells to ~7 Log_10_(CFU/mL) after one session of MB + KI-aPDT killing (Figure 9). 

## 4. Discussion

The focus of this study was to delve into the optimization of four parameters for MB + KI-aPDT, using two kinds of uniform design methods, to improve the effectiveness of aPDT killing on three Candida strains. These parameters included the concentrations of MB and KI, light dose, and MB’s incubation time. To our knowledge, this is the first study to optimize MB + KI-aPDT parameters using the uniform design method, which comprehensively consideres not only each parameter individually but also the interactions among them. 

We found that within the ranges of MB concentrations of 0.1 to 1.0 mM, KI concentrations of 100 to 400 mM, light doses of 10 to 70 J/cm^2^, and MB’s incubation times of 5 to 35 min, MB + KI-aPDT killing efficiencies on three Candida species were all negatively associated with MB concentration. There was no consensus among the investigations on the optimal concentration of MB or light dose for Candida species. Rossoni et al. [31] used 0.3 mM MB pre-incubated in the dark for 5 min and irradiated with a light dose of 26.3 J/ cm^2^, which could reduce *C. albicans* cells from 0.49 to 2.34 log_10_ (CFU/mL). Souza et al. [32] used 0.1 mg/mL MB (equal to 0.3 mM) pre-incubated in the dark for 5 min, and irradiated with a light dose of 39.5 J/ cm^2^, which could reduce maximum *C. albicans* cells to 2.71 log_10_ (CFU/mL). Machado-de-Sena et al. [33] used 1 mM MB pre-incubated in the dark for 10 min, and irradiated with a light dose of 36 J/ cm^2^, which could reduce *C. albicans* cells to 1.66 log_10_ (CFU/mL). Taken together, these investigations imply that an increase in MB concentration from 0.3 to 1.0 mM may result in a decrease in the killing effectiveness of MB-aPDT (2.34 or 2.71 vs. 1.66 log_10_ (CFU/mL)). Although these studies did not add KI, they agreed with the present study’s Equations (1) and (2) from Table 5.

When employing MB + KI-aPDT, Freire et al. [25] explored the best light dose for aPDT and found that the highest log reductions of *C. albicans* cells were achieved with MB (100 µM) combined with KI (100 mM) and a light dose of 40 J/ cm^2^, whereas increasing the light dose to 60 J/cm^2^ did not enhance MB + KI-aPDT’s killing effectiveness. This is in agreement with the present study’s Equation (1) in Table 5, which shows that MB + KI-aPDT killing efficacy is negatively associated with light dose. However, they did not explore more MB concentration levels in this study.

It can be seen in Table 7 and Figure 5 that, despite all three Candida species having a negative association between MB + KI-aPDT killing efficiency and MB concentration, they have different combinations of optimal aPDT parameters from each other. The interactions between MB concentration and light dose on *C. albicans* ATCC 90028, and the interactions between light dose and MB’s incubation time on *C. glabrata* ATCC 2950, play contrary roles in MB + KI-aPDT killing effectiveness. 

The mechanism of interactions between MB concentration and light dose has yet to be elucidated. The present study increased light dose by prolonging irradiation time. As we know, MB degradation increases as irradiation time is prolonged, along with a decrease in MB concentration. Matveev et al. [35] used optical absorbance to estimate MB concentration, and found that aqueous MB solutions have a nonlinear behavior of optical absorbance, suggesting the formation of MB dimers and trimers in the specific concentration range. MB monomers and dimers may be involved in different kinds of photodynamic reactions, and their concentration influences the mechanisms and types of ROS produced [36]. The MB monomer produces ^1^O_2_ by photochemical pathway type II, in which energy is transferred to the oxygen molecule from the triplet excited state of MB (^3^MB^+^). While higher MB concentrations of MB dimer [(MB)_2_^2+^] produce semi-reduced MB radicals (MB*), they are oxidized to produce superoxides, including O^2−^, H_2_O_2_, and HO• by photochemical pathway type I [36]. Hence, the nonlinear behavior of MB concentration, combined with a prolonged irradiation time leading to photobleaching, constantly changes the ratio of MB monomers and polymers, as well as the types and quantities of ROS. This dynamic relationship between MB concentration and light dose may subsequently influence the effectiveness of aPDT in killing microorganisms.

With regard to the interactions between light dose and MB’s incubation time in *C. glabrata* ATCC 2950, this could be caused by distinct Candida cell walls. Candida species generally exhibit a high degree of cell surface hydrophobicity [7]. In contrast, MB is highly water-soluble and has poor cell/tissue penetration [37]. Nevertheless, morphological analysis reveals that the cell membrane of *C. albicans* is the target of MB-aPDT [38]. This means that MB can bind to the Candida cell and penetrate through the outer cell wall to reach the plasma membrane or the cytoplasm. Membrane damage and subsequent increased permeability, alteration of cytoplasmic membrane proteins, disturbed cell wall synthesis, and potassium ion loss are suggested causes of cell death [39]. Since ROS produced during aPDT have very short lifetimes and a limited diffusion distance, they can only kill pathogens that are located in close proximity to where they are produced [40]. The Candida cell wall is the first barrier for MB to adhere to and penetrate through. The Candida cell wall consists of an inner layer of polysaccharides (chitin coated by 1,3-β-glucans and 1,6-β-glucans) and an outer layer of proteins glycosylated with mannan [41]. *C. glabrata* has lower levels of cell wall β-glucan compared with *C. albicans*, but the mannoprotein content is a 50% higher than that of *C. albicans* [42]. The coefficient of the interactions between light dose and MB’s incubation time in *C. glabrata* ATCC 2950 was negative. This indicates that when MB’s incubation time is increased, light irradiation time is shortened compared with when MB’s incubation time is lower. This correlates to the characteristics of the *C. glabrata* cell wall. The denser-packed mannoproteins in the outer layer may result in slower penetration of MB. Elevating the incubation time of MB was necessary for MB to adhere to and penetrate through the *C. glabrata* cell wall adequately. Once ROS are produced during aPDT and the outer layer is disrupted, the thinner glucan network makes the subsequent cell death mentioned above easier and quicker.

Apart from the Candida cell wall, the reason why three Candida species have different combinations of optimal aPDT parameters from each other could be their intrinsic metabolic determinants. According to previous literature, different members of the same class of microbes seem to have different susceptibility patterns to aPDT due to intrinsic metabolic determinants, such as enzymatic defense against reactive oxygen species and efflux pumps that recognize photosensitizer molecules, etc. [43]. It was reported [44] that *C. glabrata* exhibits intrinsically high H_2_O_2_ resistance compared with *C. albicans*, which correlates with higher whole-cell catalase activity. H_2_O_2_ is one type of superoxide produced by the photochemical pathway type I during aPDT, as mentioned earlier. *Candida* species also express certain cell wall proteins (CWPs) that aid in adhering to and colonizing human host cells, as well as adapting to ROS generated by phagocytic cells of the human host [45]. Amongst *Candida* species’ CWPs, the moonlight-like CWPs are regarded as the first line of defense against ROS [45]. Medrano-Diaz et al. [46] proved that moonlighting proteins, which induced protection in a mouse model against Candida species, are different in *C. albicans* and *C. glabrate*. Ramirez-Quijas et al. [45] demonstrated that the moonlight-like CWPs are differentially regulated in *C. albicans*, *C. parapsilosis*, and *C. glabrate*. Accordingly, the MB + KI-aPDT killing efficacy on these three Candida species may also be altered. 

Additionally, ABC (ATP-binding cassette) and MFS (major facilitator superfamily) exporters are two efflux systems in Candida species [47]. MB also behaves as a substrate of yeast efflux systems [43]. ABCs and MFSs are both involved in MB export. Overexpression of both systems could reduce MB uptake, as well as the killing effect of MB-mediated aPDT on *C. albicans* [43]. Little is known about the MB export in *C. glabrata* or *C. parapsilosis*. However, similar to MB as a substrate, azoles have been found to be substrates of Candida strains. MFSs and ABCs both contribute to azole resistance in Candida strains, as they can reduce the uptake of azoles, but none of the MFS proteins in *C. glabrata* have been shown to be associated with azole resistance [48]. This may imply that the amount of MB that enters the cell membrane of *C. glabrata* is different from that of other Candida strains, thereby diversifying the efficacy of aPDT as a fungicidal treatment. However, the specific diversities still need further investigation in the future.

It is worth noting that the optimal quantitative values of Y (represented by MB + KI-aPDT killing cells (log_10_(CFU/mL)) of three Candida strains), as shown in Table 7, were only calculated numbers inferred from the regression equations. The regression equations were primarily derived to determine the optimal MB + KI-aPDT parameters, rather than to obtain the exact killing log_10_(CFU/mL), as they may introduce some bias compared with the actual situation.

In conclusion, the uniform design method can optimize the combinatorial parameters of MB+KI-aPDT to achieve the most effective aPDT fungicidal effects on Candida species. The uniform design method has several advantages. Firstly, it can comprehensively consider each parameter and the interactions among them. Secondly, it can explore several levels of different aPDT parameters with the fewest number of experiments. Therefore, this approach has potential for further investigation of additional combinatorial parameters in aPDT, particularly with regard to the use of new photosensitizers for aPDT or the application of established photosensitizers in aPDT across a range of microorganisms in upcoming studies.

## 5. Conclusions

The uniform design method can be used to optimize the combinatorial parameters of aPDT, including concentrations of MB and KI, light dose, and the incubation time of MB, to improve fungicidal effects on Candida species. This method can not only apply to explore MB + KI aPDT parameters but also other photosensitizers on different pathogens in the future.

## Figures and Tables

**Table 1:** The U_7_(7^4^) uniform design table for aPDT parameters.

Parameters	Levels						
	1	2	3	4	5	6	7
X_1_ = MB (mM)	0.10	0.25	0.40	0.55	0.70	0.85	1.00
X_2_ = KI (mM)	100	150	200	250	300	350	400
X_3_ = Light dose (J/cm^2^)	10	20	30	40	50	60	70
X_4_ = incubation time (min)	5	10	15	20	25	30	35

**Table 2:** The U_7_(7^4^) uniform design method’s corresponding parameters assignment table.

Detection Schemes	Parameters and Levels			
	X_1_ = MB (mM)	X_2_ = KI (mM)	X_3_ = Light Dose (J/cm^2^)	X_4_ = Incubation Time (min)
A	0.10	150	30	30
B	0.25	250	60	25
C	0.40	350	20	20
D	0.55	100	50	15
E	0.70	200	10	10
F	0.85	300	40	5
G	1.00	400	70	35

Notes: X_1_, X_2_, X_3_, and X_4_ represent MB concentration, KI concentration, light dose, and MB’s incubation time, respectively.

**Table 3:** The U_6_(6^4^) uniform design table for aPDT parameters.

Parameters	Levels					
	1	2	3	4	5	6
X_1_ = MB (mM)	0.05	0.06	0.07	0.08	0.09	0.10
X_2_ = KI (mM)	50	60	70	80	90	100
X_3_ = Light dose(J/cm^2^)	5	6	7	8	9	10
X_4_ = incubation time (s)	50	100	150	200	250	300

**Table 4:** The U_6_(6^4^) uniform design method’s corresponding parameters assignment table.

Detection Schemes	Parameters			
	X_1_ = MB (mM)	X_2_ = KI (mM)	X_3_ = Light Dose (J/cm^2^)	X_4_ = Incubation Time (s)
H	0.05	60	7	300
I	0.06	80	10	250
J	0.07	100	6	200
K	0.08	50	9	150
L	0.09	70	5	100
M	0.10	90	8	50

Notes: X_1_, X_2_, X_3_, and X_4_ represent MB concentration, KI concentration, light dose and MB’s incubation time, respectively.

**Table 5:** The regression models for MB + KI-aPDT and MB-aPDT efficiency obtained by the U_7_(7^4^) uniform design method.

Equations	Strains	Regression Model	Model *p* Value	Model Fit
(1)	*C. albicans* ATCC 90028	Y = 7.126 + 1.199X_1_X_3_ − 1.742X_1_^2^ + 0.206X_2_^2^ −0.361X_3_^2^ (MB + KI-aPDT)	<0.001	R = 0.959, adjusted R^2^ = 0.913
(2)	*C. albicans* ATCC 90028	Y = 5.392− 0.743 X_1_ + 0.361 X_3_^2^ (MB-aPDT)	<0.001	R = 0.771, adjusted R^2^ = 0.575
(3)	*C. parapsilosis* ATCC 22019	Y = 10.724 − 0.867X_1_ − 1.497X_2_ + 0.560X_3_ + 1.298X_2_^2^ (MB + KI-aPDT)	<0.001	R = 0.987, adjusted R^2^ = 0.968
(4)	*C. parapsilosis* ATCC 22019	Y = 10.577 − 3.237X_1_ + 1.075 X_3_^2^ + 2.836X_1_^2^ − 0.876X_1_X_3_ − 0.401X_4_^2^ (MB-aPDT)	<0.001	R = 0.987, adjusted R^2^ = 0.966
(5)	*C. glabrata* ATCC 2950	Y = 0.892 − 0.956X_1_ + 2.296X_3_ + 1.299X_4_^2^ −3.316 X_3_X_4_ (MB + KI-aPDT)	<0.001	R = 0.914, adjusted R^2^ = 0.769
(6)	*C. glabrata* ATCC 2950	Y = 13.04 − 3.809X_1_ + 3.674X_1_^2^ + 1.038X_3_^2^ −1.172X_1_X_3_ − 0.402X_4_ ^2^(MB-aPDT)	<0.001	R = 0.994, adjusted R^2^ = 0.984

Notes: aPDT fungicidal efficiency is defined as Y; X1, X2, X3, and X4 represent MB concentration, KI concentration, light dose, and MB’s incubation time, respectively.

**Table 6:** Regression models of MB + KI-aPDT efficiency obtained by the U_6_(6^4^) uniform design method.

Equations	Strains	Regression Model	Model *p* Value	Model Fit
(7)	*C. albicans* ATCC 90028	Y = −0.997 + 0.635X_2_X_3_	0.001	R = 0.635, adjusted R^2^ = 0.376
(8)	*C. parapsilosis* ATCC 22019	Y = −1.727 + 0.707X_2_X_3_	<0.001	R = 0.707, adjusted R^2^ = 0.478
(9)	*C. glabrata* ATCC 2950	Y = −1.511 + 0.559 X_2_X_3_ + 0.457X2X_4_	<0.001	R = 0.812, adjusted R^2^ = 0.627

Notes: aPDT fungicidal efficiency was defined as Y; X_1_, X_2_, X_3_, and X_4_ represent MB concentration, KI concentration, light dose, and MB’s incubation time, respectively.

**Table 7:** Combinations of the optimized MB + KI-aPDT parameters on Candida strains obtained by the U_7_(7^4^) uniform design method.

Strains	X_1_ = MB Concentration (mM)	X_2_ = KI Concentration (mM)	X_3_ = Light Dose (J/cm^2^)	X_4_ = Incubation Time (min)	Y = MB + KI-aPDT Killing Efficiency (Log_10_(CFU/mL))
*C. albicans* ATCC 90028	0.1	400	20	5	16.322
*C. parapsilosis* ATCC 22019	0.1	400	70	5	67.08
*C. glabrata* ATCC 2950	0.1	100	10	35	42.671

Notes: Y, X_1_, X_2_, X_3,_ and X_4_ represent the killing efficacy of MB + KI-aPDT on three Candida strains (Log_10_(CFU/mL)), MB concentration, KI concentration, light dose, and MB’s incubation time, respectively.

## Data Availability

The data presented in this study are available on request from the corresponding author. The data are not publicly available due to privacy restrictions.

## Abbreviations

aPDT	antimicrobial photodynamic therapy
MB	methylene blue
KI	potassium iodide
*C. albicans*	*Candida albicans*
*C. glabrata*	*Candida glabrata*
*C. parapsilosis*	*Candida parapsilosis*
MRSA	methicillin-resistant *Staphylococcus aureus*
ROS	reactive oxygen species
LED	light-emitting diode
ATCC	American Type Culture Collection
PBS	phosphate-buffered saline
PDA	potato dextrose agar
SDA	Sabouraud glucose agar with chloramphenicol
CFU	counting forming unit
ABC	ATP-binding cassette
MFS	major facilitator superfamily
